# International Summer School, ‘ From Genome to Life’

**DOI:** 10.1002/cfg.222

**Published:** 2002-12

**Authors:** Jo Wixon

**Affiliations:** MRC UK HGMP Resource Centre Genome Campus Hinxton Cambridge CB10 1SB UK

## Abstract

This report from the International Summer School ‘From Genome to Life’, held at the Institute d'Etudes Scientifiques de Cargèse in Corsica in July 2002, covers the talks of the invited speakers. The topics of the talks can be broadly grouped into the areas of genome annotation, comparative and evolutionary genomics, functional genomics, proteomics, structural genomics, pharmacogenomics, and organelle genomes, epigenetics and RNA.


**Comparative and Functional Genomics**
*Comp Funct Genom* 2002; **3**: 535–550.
Published online in Wiley InterScience (www.interscience.wiley.com). **DOI:** 10.1002/cfg.222

**Feature**

### Meeting Highlights: International Summer School, 'From Genome to Life'

Institute d'Etudes Scientifiques de Cargèse, Cargèse, Corsica, France, 15–26 July 2002

Jo Wixon*, Managing Editor
*MRC UK HGMP Resource Centre, Genome Campus, Hinxton, Cambridge CB10 1SB, UK*

*Correspondence to:
Jo Wixon, MRC UK HGMP Resource Centre, Genome Campus, Hinxton, Cambridge CB10 1SB, UK.
E-mail: jwixon@hgmp.mrc.ac.uk*

### Abstract

**This report from the International Summer School 'From Genome to Life', held at the Institute d'Etudes Scientifiques de Cargèse in Corsica in July 2002, covers the talks of the invited speakers. The topics of the talks can be broadly grouped into the areas of genome annotation, comparative and evolutionary genomics, functional genomics, proteomics, structural genomics, pharmacogenomics, and organelle genomes, epigenetics and RNA. Copyright © 2002 John Wiley & Sons, Ltd.**

### Genome annotation

Steve Brenner (University of California, Berkeley, USA) explained **the genome annotation challenge** that faces all genome sequencers and those interested in functional genomics. He pointed out that it is common for only ~20% of genes in a genome to have been studied experimentally prior to sequencing, and the situation is getting much worse for some of the bacterial genomes currently under way. He also pointed out that functional prediction (although it has been claimed to be solid in some cases) cannot be certain unless it is experimentally verified. Taking the example of *Mycoplasma genitalium*, he has compared annotations by TIGR, GeneQuiz (two versions) and Eugene Koonin. He does see some compatible annotations, although some have subtle differences (such as just one method spotting a paralogue or an incomplete gene). In some cases one method may use the term hypothetical, another makes an assumption from the best sequence match. For some genes two or more methods agree, but in several cases the results are irreconcilable. Assuming that cases of agreement are correct, the mean error rate is ~8%, but he guesses that the actual error rate is around 20%. The errors can arise from poor sequence comparison (not true homology), incorrect inferences from homology, and propagation of erroneous data. He contrasted SWISS-PROT, which guards against propagation of error and incorporation of bad data but which does not give the source of its annotation, and GenBank, which is filled with errors but does at least give the source of the data, enabling users to check it for themselves. He did, however, point out that, on occasion, confusing cases where annotation methods disagree can uncover novel protein functions. Amongst a range of proteins that were predicted as nuclear proteins by one tool and as membrane proteins by another, they identified 53 that had both transmembrane regions and DNA-binding domains, making them potential membrane-tethered nuclear proteins. Many of these are nuclear steroid receptors. They all have a hypothetical cleavage site that would separate the transmembrane regions from the DNA-binding domains. Expression of whole and truncated (DNA-binding domain only) protein for several examples has demonstrated membrane and nuclear localization, respectively, as expected.

Hugues Roest-Crollius (Genoscope, Evry, France) presented a summary of the current progress in ***Tetraodon nigroviridis*** **sequencing and annotation**. In collaboration with the Whitehead Institute Center for Genome Research (WI), the Genoscope team have sequenced 8.3 genome equivalents of

Copyright © 2002 John Wiley & Sons, Ltd.

*Tetraodon* (40% WI, 60% Genoscope), a preliminary assembly of 5.6 equivalents was released in May. The assembly is based on in-house software, which first clusters the sequence reads using a fast algorithm, and then these clusters are provided to Phrap for assembly. Phrap contigs are then fused, when possible, and organized into scaffolds. This latter assembly has been used as the basis for a preliminary annotation. The annotation is based on Exofish and other tools (including Genewise) and resources (such as a large set of *Tetraodon* cDNAs). He presented examples of genes that are particularly compact, and of genes that are difficult to annotate. The most 'compact' gene in their annotation appears to be the phenylalanine tRNA synthetase, which is about 340 times smaller in *Tetraodon* than in human. The Exofish approach (exon finding by sequence homology) identifies regions that are conserved in *Tetraodon* and human, the alignments falling in coding regions are then used to build 'ecores'. Then he presented an update of the count of human genes based on ecores from the near-fully sequenced human and *Tetraodon* genomes. Their new estimate stands at ~26 000 genes and ~5000 pseudogenes. He pointed out that this is in line with recent findings from projects such as Ensembl but, interestingly, still lower than many researchers are prepared to accept. Finally, he summarized the status of the *Tetraodon* physical mapping project. The current map is based on ~27 000 fingerprinted BAC clones and 2300 markers mapped by hybridization. FISH mapping is under way to anchor the map onto the *Tetraodon* chromosomes.

Gwennaele Fichant (LCB, Marseilles, France) presented one partial answer to the annotation problem for bacterial genomes, describing an approach for **identifying, assembling and classifying integrated biological systems from completely sequenced genomes**, which has been tested on ABC transporters. This *in silico* reconstruction relies on an understanding of the components of ABC systems. Exporters are made up of two membrane-spanning domains (MSDs) and two nucleotide-binding domains (NBDs), whereas importers have the same structure but with an additional soluble, or membrane-anchored, solute binding protein (SBP). In eukaryotes there is one gene per transporter, but in prokaryotes each component is encoded by a different gene. NBDs show high sequence conservation, in particular of three motifs, the MSD superfamily shows fuzzy, global conservation, with 4–8 transmembrane regions, and the solute binding proteins show no conservation — they are secreted proteins with signal peptides. Fortunately, the genes are often found together in bacterial genomes with operon structure. If they are not in the same operon, they are usually close by, on a split or neighbouring operon. Their first project was *Bacillus subtilis*, which had very few known ABC systems. They found 78 NBDs and 103 MSDs, some of which had the two parts as a fused gene, and 37 SBPs. Looking for compatible subfamilies of NBDs and MSDs (e.g. both with homology to sugar importers) and building phylogenetic trees of the genes allowed them to reconstruct 59 complete systems, 10 lone NBDs, two lone SBPs and 11 pairs of MSDs with an SBP. Some NBDs are involved in more than one system. Since then they have applied the approach to a wide range of fully sequenced genomes, of bacteria, archaea and *Saccharomyces cerevisiae*, the data are available at **http://ir2lcb.cnrs-mrs.fr/**

### Comparative and evolutionary genomics

Olivier Gascuel (LIRMM, Montpellier, France) gave an overview of **phylogeny reconstruction from sequence data**, explaining the basic principles behind the popular approaches and comparing them.

Phylogenetic approaches are based on multiple alignments of evolutionarily related sequences and assume that the changes observed come from simple mutation mechanisms, such as substitutions, insertions and deletions. Once the alignment has been made, gaps are eliminated, identifying blocks of homology. The alignment is very important to the end result; a small change in the alignment can hugely affect the phylogenetic tree result. The alignment is used to generate a matrix of differences between the sequences, which can then be used to build trees.

The parsimony approach is the oldest method. It aims to identify the most parsimonious tree, which is the one in which the fewest events have to be induced to explain the multiple alignment. It assumes that multiple substitutions are rare and uniformly distributed. It is easy to calculate the parsimony of a tree but there are many possible trees, which makes identifying the best tree

Copyright © 2002 John Wiley & Sons, Ltd. 

hard, so heuristic algorithms are usually applied to find the best tree. He gave an example, describing DNAPARS, which inserts new sequences into a growing tree in positions that give the most parsimony, then improves the tree by tree swapping, based on reducing parsimony. Modern models allow for transitions and transversions and different nucleotide frequencies, and stochastic models can be used to deal with multiple substitutions. This approach can deal with 200–300 taxa.

Distance-based methods compute a matrix of pairwise evolutionary distances between the sequences and build a tree based on the matrix, using a heuristic approach. This can use the least squares method, neighbour joining or the minimum evolution method. It is possible to assign different costs to different mutations and to make correction for multiple changes at the same position. It is also possible to allow for different base compositions and different rates of base change. Examples of tools of this type are ADD TREE, NJ, BioNJ and Neighbor.

In the maximum likelihood approach, a model of sequence evolution is chosen (which can be simple or complex) assuming that all sites evolve independently. The likelihood of the trees is then computed, based on the multiple alignment, and the likelihood is maximized. This powerful method is much slower than distance or parsimony methods, but usually works better in simulation tests. It can cope with 100 taxa at best.

He concluded by highlighting some important points to bear in mind; that gene and species trees can be very different; the effects of horizontal transfer of genes on the meaning of trees; and the difference between duplication and speciation (paralogues vs. orthologues).

Edward Trifonov (Weizmann Institute, Israel), gave a presentation entitled '**Early evolution: from first codons to first proteins**'. He asked whether all the amino acids were present to start with and, if not, which ones were first, those which were more stable, or those with the most stable codon/anticodon pairs? To answer this, he made a set of vectors to define the opinions of researchers looking at the order of evolution of amino acids (allowing for agreements and citations). Looking at the mean ranking of the amino acids — G, A, D, V, P, S, E, T, etc. — nine of the 10 abiotic amino acids identified by Miller were first, and at the bottom of the table were the amino acids with the loosest codon assignment across species. This showed that the first triplets were the most stable ones, GGC and GCC. New ones were formed by single base changes from these and appeared as complementary pairs. As one might expect, looking at conserved stretches between bacteria and eukaryotes does detect a bias towards Gly, and comparing eubacteria against the others highlights the overall chronology. The chronology suggests that the earliest oligopeptides were mosaics of residues from two independent amino acid alphabets, with the elementary mosaic unit being six residues long. He proposes that the next stage of early protein evolution was to form closed loops of chains, which would require 20–40 amino acids. This size of loop is commonly detected in protein crystal structures. He has derived prototype sequences and secondary structures of the ancestral proteins and mapped them onto known proteins and structures (for further information on this work, see Berezovsky and Trifonov in this issue, p. 525).

In his talk '**Origin and evolution of DNA and DNA replication mechanisms: the viral connection**', Patrick Forterre (IGM, Université Paris Sud, Orsay, France) discussed the growing evidence that essential enzymatic activities for the production and replication of DNA have been invented more than once. Archaeal proteins for DNA replication are more like those of eukaryotes, despite the mechanism being more like that of bacteria. Looking at *Pyrococcus*, they saw that the standard route of uracil metabolism is not used in synthesis of archaeal DNA; ThyA, the crucial enzyme for the last step of the pathway where dUMP is converted into dTMP, is absent. *Pyrococcus* appears to have an entirely different, flavin-dependent mechanism for thymidylate synthesis. Other critical enzymes in DNA replication appear to have been invented twice, e.g. the Archaea have an evolutionarily unrelated family of type II topoisomerases, which could have been gained from viruses. He then went on to speak about the switch from RNA to DNA genomes. DNA is more stable and has more reliable replication, but these alone cannot explain the selection of the first DNA organism. He proposed that DNA appeared in the context of the competition between early RNA cells and viruses, suggesting that DNA was selected as being RNAse-resistant in more than one independent event, and the various DNA metabolism enzymes

Copyright © 2002 John Wiley & Sons, Ltd. 

and replication mechanisms then arose in the different lineages. The cells could then use RNAse to combat viruses. However, viruses then changed to U-DNA, to evade the RNAse, and eventually to T-DNA, with the invention of thymidylate synthase. He also commented that evidence indicating that some eukaryotic nuclear-encoded genes have come from viruses has recently come to light. He feels that viruses have played an important role in evolution and suggested that plasmids may have originated from viruses.

André Goffeau (ENS, Paris, France) presented **a phylogenetic classification of yeast membrane proteins**, in particular the transporters. He uses an adaptation of the transporter classification system established by Milton Saier [5] to make groupings based on phylogenetic data. The system uses a five-digit code; the first two digits are used to represent the class of transporter based on its mechanism, e.g. 1 is for channels or pores, 2 is for permeases (electrochemical potential-driven transporters), and 2A covers the porters — uniporters, symporters and antiporters — of which there are 77 families. Class 9 is for incompletely characterized transporter systems, and he has initiated the use of a class 10 for membrane proteins that are not transporters. The next two digits represent the phylogenetic data, identifying the superfamily and subfamily membership. The final digit is used to identify the substrate of the transporter. He uses TMHMM to predict transmembrane proteins and classifies anything with two or more predicted transmembrane spans. When a new protein cannot be assigned with high certainty, an 'x' is used to indicate unproven family, 'y' to indicate unproven subfamily, and 'z' for substrate unknown. Third digits (superfamily) are assigned if a protein shows a 10% identity with a probability of $E < 10^{-20}$; fourth digits (subfamily) are assigned if a protein has 20% identity with a probability of $E < 10^{-35}$; and fifth digits (substrate) at 35% identity with a probability of $E < 10^{-65}$. These are arbitrary scores but they have worked for the genes they have found so far. His analysis of the *S. cerevisiae* genes using this phylogenetic approach has identified new families requiring new classifications. It also highlighted outliers, unusual genes which are of further interest. Looking at the recent Genolevures data from 16 yeast genomes, he has identified 45 transporter families, including new transporters not found in *S. cerevisiae*, which are mainly permeases.

Bernard Labedan (IGM, Université Paris Sud, Orsay, France) discussed the merits of taking **a genomic approach to molecular evolution**. He contrasted the different approaches to building a phylogeny. The classical approach is to look at just one gene, across a range of species. Dayhoff suggested using ancient paralogues to allow outgroups to be made, which help to root this type of tree. The tree obtained cannot be used to generate a species tree, but is useful for deducing the history of that protein. Another approach is to compare all the proteins of one organism (intragenomic). This allows the detection of all paralogues and all the modules shared by proteins. His study of *Escherichia coli* has shown that duplication and gene fusion are important mechanisms of protein evolution, and allowed him to predict the repertoire of modules that would have been present in the ancestor of *E. coli* (for more details on this work, see Zouine *et al.*, in this issue, p. 493). He then discussed the intergenomic approach, which is an exhaustive comparison of all genes across organisms. He presented his studies comparing *E. coli*, *H. influenzae*, *H. pylori* and *C. jejuni*, searching for all paralogues and all orthologues. *E. coli* has the biggest proportion of species-specific genes, and has more small families of paralogues, which he thinks may be needed for survival in adverse conditions. From the comparison he deduces that the last common ancestor of these bacteria had 68.5% of genes that were unique in that genome and had no orthologues in other species. The paralogous genes that had orthologues in other species are the class of gene that has expanded the most since the last common ancestor. Finally, he discussed the phylogenomic approach, which is based on the observation that there is an inverse relationship between the phylogenetic distance between two species and the evolutionary distance separating their orthologues from their paralogues. He used this to estimate the phylogenetic distances for each pair of bacteria. After calculating the distances, he took the means and then made a matrix of the means of means, which he used to build a tree of 56 prokaryotic genomes. The overall grouping of the bacteria was unchanged whether he used small or large gene families, but the specific order did change. Since the larger families tend to be crucial genes across species, whereas the smaller ones tend to more specific and can be due to lateral transfer, he feels that using the larger families could deal with lateral transfer better.

Copyright © 2002 John Wiley & Sons, Ltd. 

Eugene Koonin (NCBI, NIH, Bethesda, MD, USA) spoke about **making evolutionary inferences from whole-genome comparisons**. He first outlined the history of phylogenetics, starting with the work in the 1960s of Zuckerhandl and Pauling, who made molecular phylogenetic trees of cytochrome *c* and globin. They believed that a true tree could be built if one had enough sequences. In the 1980s, Carl Woese made his rRNA trees producing the standard model of archaea, bacteria and eukaryotes. He felt that a detailed tree would be possible if phylogenetic methods were properly refined. In recent years, W. Ford Doolittle's work has uncovered horizontal gene transfer, which threatens to uproot the tree of life.

Koonin constructs and analyses clusters of orthologous groups (COGs), which represent ancient conserved families of proteins. Currently, the resource covers 58 complete genomes (11 archaea, 46 bacteria and one unicellular eukaryote), grouping 105 816 proteins into 4075 COGs. The majority of prokaryote proteins belong to COGs, but only one-third of yeast proteins do. Most of the COGs are represented in only a small number of clades, providing evidence that horizontal gene transfer (HGT) and clade-specific gene loss are important evolutionary mechanisms. He has produced algorithms to calculate the IQ of each COG, which is the minimum number of events (gene loss, emergence, or HGT) needed to reconcile the phylogenetic pattern of a COG with the topology of the species tree. The inconsistency of the COGs with the topology of the species tree indicates the pervasiveness of gene loss and HGT; fewer than 10% follow the tree with no inferred events.

He has explored potential approaches to constructing genome trees of prokaryotes. Building a matrix of the presence/absence of orthologues, and using this to calculate the least distance to the common ancestor to form a tree, gave grouping of bacterial parasites and was not like the tree of life; it seemed to primarily reflect gene loss. Looking at conservation of gene order (i.e. genes that are adjacent in one or more genomes, and separated by less than three genes in two or more other genomes) and generating a presence vector to give a tree put the proteobacteria together and the Gram-positives together and looked more like the phylogenetic tree, combining the phylogenetic signal with HGT. Using the median distance between orthologues gave a tree more like the phylogenetic tree, in which some interesting new clades emerge. Making a concatenated alignment of 32 ribosomal proteins on which to base the tree gave a tree like the phylogenetic tree, with a stronger signal and some new clades. As a result of this, he identified three potential new clades: *Aquifex/Thermotoga*, *Deinococcus/Mycobacteria/Synechocystis* and *Spirochaetes/Chlamydia*. Taking a census of trees for multiprotein families with wide phylogenetic distances and low numbers of paralogues, he checked the reliability of these clades by seeing how many competing topologies there were, and how many multiple protein families agreed with the clades. Each test supported the three new clades.

He concluded by saying that he feels that the concept of a tree of life still makes sense, but it should not be construed as a full, accurate depiction of organismal evolution, but rather as a central trend, which may not apply to the majority of genes. He thinks that methods based on a more or less traditional analysis of large sets of well-selected genes (minimally subject to HGT) can be informative.

Hiroyuki Ogata (IGS, Marseilles, France) spoke about a **comparative genome analysis of *Rickettsia***. His group have compared the complete genome of *Rickettsia conorii* (which is carried by ticks and causes Mediterranean spotted fever in humans) with that of *R. prowazekii* (which is carried by lice and causes typhus). There are significant differences between the genomes of the two species; *R. prowazekii* has just 834 ORFs and less than 50 repeats, whereas *R. conorii* has 1374 ORFs and around 650 repeats. *R. conorii* has actin-based motility and is not sensitive to penicillin or erythromycin, unlike *R. prowazekii*. The comparison shows almost complete co-linearity between the two genomes, which makes it easy to identify orthologues. Several genes appear to be split in one or the other genome, some of which appear to be able to make functional proteins. There are 30 *R. prowazekii*-specific genes (six with remnants in *R. conorii*) and 552 *R. conorii*-specific genes (229 of these have remnants in *R. prowazekii*). A *R. prowazekii* annotation by the Koonin group identified 16 genes obtained from the host, 10 of animal origin, four from fungi, one from plants and one of unknown origin; he suggested that these might have come from transfer from the ancestor of *Chlamydia*, which has 15 genes of plant origin.

Copyright © 2002 John Wiley & Sons, Ltd. 

Their analysis also identified a *Rickettsia* palindrome element (RPE). These ~150 bp-long stretches would form hairpin loops of secondary RNA structure; 23 of the 45 copies in *R. conorii* and nine of the 10 in *R. prowazekii* are in ORFs, which might imply that these are pseudogenes, except for the fact that several have crucial functions. They can find no evidence of common function for the genes with the palindromes, but in all cases the insertions are located in external loops of the protein structure and do not appear to disrupt functional sites. His group are currently carrying out experiments to check that these genes encode functional proteins. The RPE sequence evolves faster than the host gene and the palindrome structure decays after insertion.

### Functional genomics

Titia Sijen (Hubrecht Laboratory, Utrecht, The Netherlands) spoke about the mechanism of **RNA interference and transposon silencing in *Caenorhabditis elegans***. RNA interference (RNAi) is a dsRNA-induced post-transcriptional mechanism of repression of expression of genes. To use RNAi to knock down a gene of interest in *C. elegans*, it is possible either to inject dsRNA of the gene into the worms or have them express it from a transgenic array, or soak the worms in a solution of dsRNA, or feed them *E. coli* expressing the dsRNA. The dsRNA is 'diced' into 22 bp-long siRNA (these are a common factor in the mechanism in other species) that direct the degradation of the mRNA of that gene. RNAi is inherited by progeny in a process which includes an amplification step, which must be performed by an RNA-directed RNA polymerase. *C. elegans* has four homologues, mutations in all of which affect RNAi in various ways. Although transposons move around in somatic cells, this is blocked in the germ line. The presence of transposon siRNA has been demonstrated in germ line cells, suggesting that RNAi is the biological method of defence against transposons. Large-scale RNAi studies have been performed in the worm using a library of *E. coli* expressing 87% of *C. elegans* genes. The results of the phenotypic analyses are available from Wormbase.

In plants, RNAi is a defence against viral RNAs; it is possible to make plants resistant to viruses by giving them viral dsRNA. This could also be exploited for safely suppressing selected genes, e.g. to delay fruit softening in tomato. In mammalian cells there have been problems with aspecific responses to the dsRNA, but this has recently been overcome by using small siRNAs. RNAi is now being used for gene knockdown for functional studies, and could be a potential future avenue for gene therapy against gene specific transcription. There are already groups looking at using it against HIV.

Karin Van de Sande (University of York, UK) presented an overview of the status of **functional genomics in plants and the GARNET project**. In the UK, the BBSRC's Investing in Gene Function (IGF) program is funding studies of *Arabidopsis*, brassicas and cereals. Within Europe, the UK, France and Germany share interests in *Arabidopsis* and cereals, amongst other plants, in The Netherlands work focuses mainly on *Arabidopsis*, tomato and potato, while in Sweden there is a lot of work done on poplars. The USA has significant funding of plant genomics from the USDA and the NSF; again, there is much interest in cereals and other crop plants. In Japan and China plant genomics focuses mainly on rice and lotus.

GARNET is a project for the functional genomics of *Arabidopsis*, which encourages collaborations between the UK *Arabidopsis* community. It provides a wide range of high-throughput user-driven services and makes data publicly available. There are services for transcript or metabolite profiling, metabolites, proteomics, forward and reverse genetics and bioinformatics. The transcript profiling project is resulting in a reannotation of the genome; they use genome-specific tags with less than 70% similarity to other genes (this is only possible for 70% of the genes). The metabolites team offer metabolite profiling or metabolic fingerprinting. The proteomics service offers a range of methods in terms of gels and mass spectrometry. The forward genetics service uses transposon mutant lines; they are making over 5000 in the Columbia and Landsberg erecta ecotypes. They also have lines for conditional activation. The reverse genetics team are sequencing the insertion sites of three collections of transposon insertion lines. The Bioinformatics team are building a database to bring together the data from all parts of the project and collaborating on international databases. The PlaNet project aims to create a network of European plant databases

Copyright © 2002 John Wiley & Sons, Ltd. 

(**http://mips.gsf.de/proj/planet/**). CATMA is one such European collaborative resource, for *Arabidopsis* microarrays (**http://www.catma.org/**).

Stephen Oliver (University of Manchester, UK) spoke **about the integration of functional genomics data for yeast**. He described the apparent redundancy of the yeast genome, which has several sets of genes with the same regulation and protein localization. These do not have qualitative phenotypes but should have quantitative effects. One way to measure this is to make metabolic flux measurements. Typically the enzymes in a pathway all have small flux control coefficients that sum to 1; a highly important enzyme would have a larger flux control coefficient. Of the ~6000 yeast ORFs, ~1000 are lethal upon deletion and show slow growth in the single mutant diploid (haploinsufficiency). The products of these genes are likely to be important in their pathways. Using the bar-coded deletion strains and the array of the bar codes, they are profiling the metabolic changes in these strains. The particular approach that they are using is called FANCY (functional analysis by co-responses in yeast), which assumes that when two mutants have the same co-response, they affect the same monofunctional unit. If one knows the unit in which one of the genes functions, then this can be predicted to be the functional unit of the other. They have already shown that the method can correctly group different types of respiratory mutants based on their metabolic spectra.

Then he described what he calls 'the big experiment', a microarray-based expression profiling of chemostat-controlled yeast growth phases (slow growth, transition, fast growth and stationary phase) under six conditions, including nitrogen, carbon (glucose), phosphorous and sulphur starvation. This has been used to compare the types of genes and pathways that are involved in the responses of yeast to each of these conditions. They have also compared proteomics data with transcriptome data and got a correlation coefficient of ~0.63 (if one data point was omitted). He was interested in looking at protein turnover, as he sees this as one of the missing dimensions of proteomics. By chase labelling with deuterated leucine in a leucine auxotroph, they measured the loss of label over time as an estimate of protein turnover (degradation rate). Some proteins were not degraded at an appreciable rate and they saw no correlation between protein half-life and mRNA half-life.

Finally, he spoke about GIMS, an object data model designed to collate international data on yeast DNA and genes, proteins and protein interactions, expression data, metabolic data, etc. It is currently available from **http://www.cs.man.ac.uk/img/gims/**. 'Canned' queries can be combined to build complex queries and they have already used it to look at whether interacting protein pairs are in the same compartment of the yeast cell. 99% of known complex members did share locations, and only half of yeast 2-hybrid, or HMS-PCL, or TAP identified pairs and complexes did. Their ultimate aim is a multispecies GIMS; proGIMS (a prokaryote version) is already under way.

Monique Bolotin-Fukuhara (IGM, Université Paris Sud, Orsay, France) gave a talk entitled '**Regulatory networks in the yeast *Saccharomyces cerevisiae***: control of oxygen and carbon metabolism'. In yeast, mitochondrial function is repressed when the carbon source is glucose, this must be due to signalling and regulation between the mitochondrion and the nucleus. YAP1 has been implicated in the response to oxidative stress and the HAP complex and HAP1 have been implicated in regulating the fermentation-respiration shift. Her group have studied their roles further using yeast genetics and microarrays.

YAP1 is a yeast c-jun homologue that binds to the AP1 site, and is known to have direct and indirect target genes. YAP1 deletants are viable but show hypersensitivity to oxidative stress. Using LacZ reporter gene fusions and microarrays, they have shown that during oxidative stress YAP1 upregulates genes that are scavengers of reactive oxygen species and chaperones, as could be expected, but also that it is required in normoxic conditions, as it has a role in the control of cell proliferation, like its human homologue, c-jun. They saw good correlation between the LacZ fusion data and the microarray data, but the array found more genes regulated by YAP1 than the fusion experiments.

They showed that HAP4 induces the expression of genes involved in the Krebs cycle and mitochondrial function when fermentative carbon sources are limiting or absent. They also identified genes that are negatively regulated by HAP4, including some genes involved in lipid biosynthesis. It up- or downregulates ~450 genes, many of which have upstream CAT binding sites. Several of these HAP4-regulated genes appear to be transcription

Copyright © 2002 John Wiley & Sons, Ltd. 

factors and she suggested that a systematic study of transcription factors should be a future aim, to provide data on the hierarchy of regulatory networks.

Marie Dutreix (Institut Curie, Orsay, France) presented a study **using DNA microarrays to observe the cellular consequences of growth in continuous exposure to low doses of ionizing radiation**. At exposures of ∼200 mGy/h, yeast cells get blocked in $G_2$ phase for some time, but do eventually restart growth. At ∼20 mGy/h cells show recombination repair and mutagenesis. Her group have looked at exposures lower than this to see what effects there are when growth is apparently normal. They found that they could use the array to see very low-level effects; even at the 0.5 mGy time point they could see small changes in gene expression compared to controls. This does depend on the gene, though, as the level of variation in the data is different for each gene; some are very variable. Among those genes that were upregulated, half were of unknown function, and 10 were involved in the oxidative stress response, so perhaps the cell is detecting protein or membrane damage. They did see some similarities between the time points, but the effects of this permanent exposure are cumulative and when they look at the most upregulated genes in each case they see no overlap. Overall they saw that 69 genes were overexpressed and more than 155 genes were underexpressed during continuous low-dose exposure.

Denis Thieffry (University of Aix-Marseilles II, France) spoke about **computational integration, analysis, and simulation of genetic regulatory networks**. His group have been modelling the Gap genes involved in *Drosophila* segmentation. The Gap module has four genes, which are expressed in defined domains along the anterior–posterior axis of the embryo in response to asymmetric maternal information (the levels and distribution of three maternal products) in the oocyte. They want to achieve an understanding of the dynamic behaviour of the system as a whole. Their model (and those of others) uses a 4 × 4 grid of the genes to predict the levels of the four proteins. They tested their model to see if it could 'predict' the levels of the products correctly for known situations, and showed that it worked for loss-of-function mutants, if there was ectopic expression, or for *cis*-regulatory mutants.

They have also worked on models of the eukaryote cell cycle (in the fly) by transposing the yeast model of Novak and Tyson. They have made a matrix of the three regulatory proteins plus MPF and worked out the possible states and transitions as a graph, which they then use to make predictions. The simulations they have run did correspond to the correct succession of phases and they have also correctly simulated a selection of mutants. Their model has allowed them to deduce that of the three regulatory proteins, fizzy seems to drive the oscillations, whereas the others modify them.

### Proteomics

Thierry Rabilloud (Grenoble, France) gave **an overview of proteomics** by 2D gels and mass spectrometry. First he highlighted the scale of the problem, explaining that a simple prokaryote will have around 2500 proteins, which are highly modified, whereas humans probably have between 100 000 and 1 000 000 proteins, which are heavily modified. In addition, there can be versions of a protein with the same number of phosphorylations but in different places, which cannot be distinguished by a 2D gel. Mass spectrometry can give some information about modifications, but once you have the mass peaks, you need to have the full genome sequence of the organism to identify the protein that they come from; EST data is not enough and even mouse–human homology is not enough to recognize trypsin digest products. Modified peptides and contaminants will give peaks that do not match the predictions, which complicates the matter further. Tandem mass spectrometry allows the operator to select a peptide of interest and further fragment it to obtain its sequence (except for Leu and Ile residues), which can help.

The main problems are the huge dynamic range of proteins present (it is not possible to resolve them by any current technique), the huge range of pI (there are some very basic proteins), the very hydrophobic proteins (these need a mix of water and organic solvent) and membrane proteins (these pose great problems for 2D gel separation). He is not convinced that subdividing cells into subproteomes helps, since even mitochondrial samples still show contaminating actin. He also spoke about comparing transcriptome and proteome data. His group have compared data sets from studies of the monocyte to immature dendritic cell transition. His group have also shown that many transcripts

Copyright © 2002 John Wiley & Sons, Ltd.

were upregulated by RT–PCR (similar results were obtained by others using microarrays); however, the proteomics study found that only one of the proteins encoded by those genes showed increased expression. They then did RT–PCR on the transcripts for those proteins that did show increased expression and saw no increase in several of the transcripts and a decrease in some others.

Finally, he described the MudPIT approach, which uses a 2D microcapillary column with strong cation exchange and reverse phase resins to separate the proteins, which are then are eluted directly into a mass spectrometer. MudPIT can identify many more proteins present in a sample than can traditional proteomics, but it is not quantitative. So it depends what information is required as to which approach should be used.

Pierre Legrain (Hybrigenics, France) described how in-depth **analyses of protein networks can lead to the discovery of protein function**. He first explained that only ~500 proteins are known to be targets of drugs today, and they come from a narrow set of protein families. It is possible to implicate proteins as potential new targets, or as a lead to drop, based on which other proteins they are related to. Hybrigenics use protein interaction networks as a way of detecting relationships and shared functions between proteins; they have an internal database with 1000s of interacting partners, from which they have identified ~100 potential targets, of these they have some functional validation of ~5 proteins. In their yeast 2-hybrid approach they run one bait against many prey using a highly complex library with up to 2–3 million prokaryote gene fragments or ~10 million human gene fragments. Typically, a few to a few hundred positives are chosen as selected interacting domains (SIDs). They use a standard production process to achieve high throughput and good reproducibility; many steps in the process are automated.

Their *H. pylori* studies identified over 1500 SIDs, and they found 15 of the 16 known Ras interactions, and four novel ones with genes from the same families as known interactors, which leads them to believe that they are true interactions. Their selection of candidate proteins is computer aided; they use their PIM Builder LIMS system and prevalidation tools followed by expert analysis, including the use of their PIM Rider and Genolink tools to prioritize targets. The SIDs are mapped onto proteins and compared to the annotation of functional domains; where possible, functional categories are then assigned.

Bertrand Seraphin (CGM, Gif, France) gave a presentation entitled '**Proteome analysis and functional characterization of protein complexes using the TAP (tandem affinity purification) method**'. Several recent large-scale studies have shown that proteins rarely act alone; many are found in complexes. Typically, different strategies have been needed to purify different proteins, so he wanted to try to design a standard method that could be broadly applied. The only way to do this is to use affinity tags and a native method (mild conditions) that retains complexes. He also wanted to use only basal expression in the host, to obtain the normal form of the complex, so the method had to have strong and specific binding of the tag. The tag that was chosen is a calmodulin-binding peptide separated from two IgG binding domains of protein A by a TEV protease cleavage site. This allows a two-step purification, using IgG beads first, and then TEV protease cleavage, using calmodulin-coated beads in the presence of calcium to obtain the complex. This method drastically reduces the volume of culture needed from 300 l to 5 l. They tested it on yeast U1 snrps and other yeast complexes and even obtained some complexes that still showed activity. They have also shown that it works in other organisms, including human cells.

To test the approach for large-scale applications, they teamed up with Cellzome for a global analysis of yeast complexes. They started with the 1739 yeast genes with homologues in other eukaryotes, of which 589 have so far been successfully purified using the TAP method, and complexes were identified in 232 cases. 58% of the complexes they found were novel; in 33% of cases they identified new components of a known complex and in 9% of cases only known components of a complex were found. They have generated a network of the complexes which can be colour coded to denote the functional categories of the complexes; this did show some grouping of complexes of related function. In running gels of the complexes, they saw that the method appears to be at least partially quantitative. This data is lost in the Cellzome project as the gel is used to generate bands for mass spectrometric analysis, but he is following this up in his lab.

Michel Werner (CEA, Saclay France) has used **microarray analysis to study the adaptation of**

Copyright © 2002 John Wiley & Sons, Ltd. 

**the yeast proteome in response to cadmium**. Proteins induced by exposure to cadmium include antioxidants, heat shock proteins and chaperones. In the sulphur–amino acid metabolic pathway, there are pairs of isoenzymes that show opposite regulation by cadmium. Looking at the proteins induced by cadmium, he saw that they have reduced sulphur content (low Met and Cys), so this seems to be a sulphur-sparing mechanism. The glutathione synthesis pathway is strongly induced, suggesting that perhaps sulphur is redirected into formation of GSH to complex the cadmium. Looking at the response in more detail using microarrays, he saw induction of detoxification genes, DNA repair genes, stress response genes and nitrogen and sulphur utilization genes, and repression of RNA and other transcription, RNA transport, ribosomal proteins and translation. Comparing data from RNA and proteins, he concluded that the response to cadmium is regulated at the transcriptional level. He wondered if it could be sulphur-containing amino acid transcription factors that regulate the response. Using the chip he identified several cadmium-induced genes that were regulated by Met4p, all of which encode low sulphur content proteins. However, there are still other cadmium-induced proteins that have lower than average sulphur content and that are not regulated by Met4p, so other transcription factors mediating the response remain to be found.

### Structural genomics

Wolfgang Baumeister (Max Planck Institute fur Biochemie, Martinsried, Germany) spoke about the use of **electron tomography to visualize supermolecular architecture inside cells**. He thinks that it is likely that there is structure beyond single macromolecules in the context of the cell (at nm scale). Many interactions are too weak or transient to be studied by biochemical methods; for this, an *in situ* approach is needed. Electron tomography can give 3D images of frozen hydrated cells with 2–4 nm resolution.

The technique has great potential for visualizing macromolecules inside cells, but this application is, as yet, in its very early stages. To make a 3D reconstruction, a set of 2D projections is taken from a single specimen. However, to obtain a detailed, undistorted reconstruction, a tilt series must be taken with as wide an angular range as possible and with as many increments as possible. The problem with this is that the electron dose of the sample must be kept subcritical to make sure that radiation damage does not erase important details; a safe dose must be spread across the sample. It is also difficult to move the sample accurately by the very small increments needed to make the series. Typically, ~150 images are taken, using 97% of the safe dosage; the rest is used to correct for inaccurate movement of the sample. He showed some examples of what can be done, including a visualization of a 20S proteasome in a *Dictyostelium* cell. Other examples are a thermosome, the GroEL complex and vesicles on their way through a membrane. The method has only been applied to two eukaryotic cell types so far, *Dictyostelium* and neurons. The diameter of the cell needs to be less than 0.5 μm to obtain good results; a cell up to 1 μm diameter can be studied but for larger cells the method must be combined with cryosectioning.

Monique Marilley (University of Aix–Marseilles, La Timone, France) spoke about **using molecular modelling and atomic force microscopy in the study of genomes**. DNA structure can vary, which affects the stability and could indicate function, such as histone binding and packing. To model the structure taking into account each atom has limitations, so typically, simpler models with a reduced number of variable parameters, are used. Base pairs all have the same dimensions, which makes mapping the next base pair easier, but the twist, roll and tilt must all be taken account of. There are several existing estimates of these parameters for any one region, which do not completely agree, so important questions are how does one select the best parameters, and how can they be improved?

In atomic force microscopy a sharp tip is held above the sample on a movable, cantilever arm. Deflections caused by interactions with the sample are measured by a photodiode. This then gives 3D real-time analysis and permits access to the external envelope of objects under observation. Imaging can be done in a vacuum, in air or under liquid (the tip is submerged) and can be used to look at single molecules or populations of molecules. Image analysis yields information on the length of the molecule and the distance between the two ends; by using end labelling of DNA, its orientation

Copyright © 2002 John Wiley & Sons, Ltd.

can also be determined. She has done experiments to see if the method can be used to study planar, or non-planar, curvature of pieces of DNA. In both cases she has found that imaging under liquid works better and that her results were confirmed by gel retardation analysis. So, AFM offers a new opportunity to study DNA curvature, but the transformation from the 3D form to 2D must be taken into account. There are several approaches to looking at DNA flexibility, DNase I is used to look at bendability, and nucleosomes to look at flexibility (commonly these methods agree, except in certain regions). Crystallography can be used to look at deformability and AFM in air could be used to look at fluctuation of experimental measures. She looked at the binding of an enhanceosome to DNA. A previous ESI study had shown that the binding caused one turn of the DNA, so she used AFM in liquid to predict the most flexible regions. Their most flexible region matched the binding site of the molecule.

Herman Van Tilbeurgh (LEBS, CNRS, Gif-sur-Yvette, France) gave an overview of **structural genomics** and then spoke briefly about some existing structural genomics projects, including the one for yeast at Orsay-Gif (**http://www.genomics.eu.org/**). Current projects are either structure-directed (building dictionaries of all available protein folds, or modelling the majority of proteins in an organism) or function-directed (determining the structure of proteins of known function or predicting function from structure). Structural genomics can provide information on protein families, surface composition, active sites, ligand interactions, mutants, SNPs and conserved residues. It is clear that we need higher throughput, as we want to know about the space–time organization of molecules, alternative structures and dynamic aspects of structure. Vitkup *et al.* [6] estimated that there are 1000–3000 unique types of fold topology; PDB currently holds $\sim$300 and it generally takes data from five sequences to characterize one fold. At 4–8 Å resolution, with insignificant sequence homology it is only possible to produce *ab initio* predictions, with poor confidence. With 30% sequence similarity and 3.5 Å resolution, it is possible to obtain 80% modelling accuracy and use structure threading; this model would allow some inferences to be made. However, to make more complex inferences, the accuracy of the model needs to be around 95%. Data from members of a family can be combined to define structural motifs; these can be used to search for new members of a family. When a sequence appears to be similar to known proteins, it is possible to apply structural superimposition. If the Z score of the match is good enough, then it is likely to be a member of the family. He then gave some examples of the highs and lows of the structure–function paradigm, including the discovery of a role for cytochrome *c* in apoptosis, which had not been predicted despite a large volume of data on its structure, and the elucidation of the function of an *M. jannaschi* ATPase completely from its structure.

Technological developments have been, and will continue to be, driven mostly by the structural genomics projects. DNA cloning, expression and purification are in some cases already automated, except for fragile proteins, and there have been some improvements in crystallization. In the area of data collection, the synchrotron technology has been linked to automated crystal handling and there have also been improvements in data analysis programs. In the NMR field there have been advances in the design of spectrometers and in labelling techniques. The next challenges for structural genomics are membrane proteins, protein complexes and proving the value of the structure–function paradigm.

Dino Moras (IGBMC, Strasbourg, France) gave a talk on **a structural genomics project for orphan nuclear receptors**. Nuclear receptors (NR) have a modular structure with a highly conserved DNA binding domain and a less well conserved ligand binding domain (LBD), separated by a hinge region. They regulate transcription in three modes, repression (with a co-repressor), derepression (with a co-activator) and activation (with a mediator). The structures of some NR LBDs have been determined, and a model canonical structure is known; to detect some features of the function, however, requires high resolution. Both the solubility and the stability of these proteins cause problems, one solution being to engineer a different form of the protein. Looking at the vitamin D receptor, they made an alignment across a range of species and saw an insertion in some species, so they made two constructs and saw that one was more stable. The next stage is to find out if they are active using solution studies. One way of stabilizing the structure is to determine the structure of the receptor when it is bound to its agonist or antagonist — ideally

Copyright © 2002 John Wiley & Sons, Ltd. 

both of these structures would be determined. Their research program is targeting 26 orphan receptors (receptors with no known ligand), and they have made 70 constructs for this. They have so far achieved expression of 30% of the constructs; 16% were soluble and they have been able to purify 14. To date they have successfully grown crystals of four receptors and have structures for three. The next step is to identify the ligand of these receptors; so far they have found the ligand of $ROR_{\beta}$ and now they are looking at $ROR_{\alpha}$.

Steve Brenner (University of California, Berkeley, CA, USA) gave a talk entitled '**structural genomics: classification and analysis**' and also spoke about the structural genomics project at Berkeley. He started by pointing out the correct meaning and use of terms such as 'homology' and 'similarity'. Homology denotes an evolutionary relationship, that two sequences have a common ancestor; it cannot be measured, it can only be inferred. Similarity, on the other hand, is a measurement from which one might infer homology. Orthologues are proteins that are related purely by vertical descent (separated only by speciation). Orthology is not a statement about function and is not defined by best hits of sequence comparisons (as these will not always find the true orthologue). Paralogues are all other homologous proteins that are related by duplication.

He explained that structure is better conserved than sequence, citing haemoglobin and myoglobin as an example of two proteins with very similar structures that have been known for many years, but we have only recently been able to see the weak sequence conservation between them. The number of structures in PDB is increasing exponentially over time. He talked about the SCOP database (a hierarchical classification of structures) as a way to organize the data. The superfamily field is a key field in the database; all proteins from a superfamily will have the same core structure and other shared features such as catalytic mechanisms. He thinks there will be 1000–2000 superfamilies in total; this relatively small number means that classifying them is a realistic task that will allow us to understand all the fundamental units of proteins.

At Berkeley they have a structural genomics project that is focused on *Mycoplasma genitalium* and *Mycoplasma pneumoniae*. Their target selection excludes proteins of low complexity and membrane proteins. For the remaining proteins they define families and remove those which already have a structure model (including from another organism). They then prioritize broadly conserved families, then those specific to one of the *Mycoplasmas*, then those that are only in the two *Mycoplasmas*. Next they look for those that are easiest to characterize, with no UGA (Trp) codons, and which are easy to clone. Starting with the 677 *M. genitalium* ORFs, they have narrowed down the list to 80 priority proteins and have already published work on 16 of them. In several cases they have found a ligand bound in the structure, which has given them clues as to the function of those proteins.

Finally he spoke briefly about SCOR, a classification of RNA structures based on duplexes (**http://scor.lbl.gov**). This gives several groups of structures with varying levels of presence, such as tetraloops and diloops. Looking over the whole classification, it seems that RNA structure has far fewer options in terms of structure.

### Pharmacogenomics

Olivier Grenet (Novartis, Switzerland) spoke about **the application of genomics to drug development**. He explained that the use of genomics in the early stages of drug development, such as in drug discovery and preclinical tests, is what he would describe as 'pharmacogenomics'. The application of SNP profiling to the trials in human patients is what he would call 'pharmacogenetics'. Genomics can be used for several stages of the process, such as to drive mechanistic hypothesis, to uncover potential markers, for safety assessment of a number of markers and for re-indication of safe drugs by looking at their profiles in genomic experiments. Genomics approaches such as chip-based gene expression analysis and real-time quantitative PCR can be used as assays for markers and mechanisms of efficacy and toxicity.

They use the Affymetrix chip with 30 000 human genes in expression profiling experiments, and the Spotfire and Genespring software and resources such as GeNet to produce clusters and trees of genes based on the expression data. For example, they have profiled oestrogen-responsive and non-responsive breast cancer tumours to define markers. They also map metabolic data onto the KEGG database to look at the regulation of metabolic pathways under various conditions. They are also

Copyright © 2002 John Wiley & Sons, Ltd.

working on combining datasets and text mining, e.g. of PubMed. They are building dictionaries of terms such as gene names, disease names and function descriptors to build into a figure of genes and links between them. They then plan to look for correlations between this and their expression data.

However, chips cannot always answer questions about polymorphisms and alternative splicing or weakly expressed genes, so they also use real-time quantitative PCR. This method allows them to study chosen genes and to specifically monitor gene variants from only minute samples. Arrays cannot differentiate between genes that are highly expressed in a very small number of cells in a tissue and those that have low expression in all cells of the sample; for this they turn to *in situ* hybridization, primarily of genes of interest identified from the arrays. They can use this to look at the localization of expression and the effects of time and treatment variations.

Wim Hol (University of Washington, Seattle, WA, USA) gave a talk about **medicinal protein crystallography and structural genomics for tropical diseases**. Anti-protozoan drugs are often drugs initially targeted against other diseases or developments of existing drugs. There are possibilities to look for new drugs, perhaps targeted at metabolic pathways. Two questions to consider are: Is protein function sufficiently critical to serve as a target?; and Is the structure of a crucial protein promising as a drug target? For instance, does it have a hydrophobic pocket? He illustrated the value of knowing the structure of targets with the example of a pentamer toxin campotecin topoisomerase; a complex compound with penta-symmetry was designed which successfully binds the toxin. Structural genomics requires careful target selection, high-throughput expression, crystallization and structure determination. Key factors in selecting targets are that they be essential and sufficiently unlike any human genes, or essential and have no human homologues at all. His group are working as part of an NIH-funded project, 'Structural genomics of pathogenic protozoa' (SGPP), that has access to two synchrotrons. The project will focus on *Trypanosoma brucei*, *T. cruzi*, *Leishmania major* and relatives, and *Plasmodium falciparum*. They aim to solve the structures of a large number of water-soluble proteins and a number of membrane proteins and protein–protein complexes. Current *T. cruzi* drugs are almost useless and have bad side effects, so the need for solutions is great. At the time of his talk, the malaria genome was due in August, the genome of *L. major* was 30% complete and that of *T. brucei* only ~10% complete. The *T. brucei* genome is proving a real problem to annotate — the prediction programs don't agree on their gene predictions, so there is much work yet to be done. He also explained that many functional genomics projects won't work on parasites and they are not easily transformed, so much more work needs to be done on developing technologies and approaches for these organisms.

George Weinstock (Baylor College of Medicine, Houston, USA) spoke about **using genomics to understand bacterial pathogenesis in *Treponema pallidum***. *T. pallidum* is a spirochaete with a periplasmic flagellum and few surface proteins. It is a stealth pathogen that can disappear and reappear 10 years later in the same patient. As yet there is no *in vitro* continuous culture system for it. A relative, *T. denticola*, is an oral spirochaete associated with periodontal disease. It can be cultured *in vitro*, it is possible to make knockout mutants, and there is a vector for complementation.

The genome of *T. pallidum* is ~1 Mb and has ~1000 protein encoding genes, 485 of which are of unknown function. 48 genes are related to virulence, including Tpr proteins, haemolysins, regulators, polysaccharide biosynthesis genes, surface proteins and host interactors. He feels that there are many more virulence genes amongst those of unknown function. They are expressing *T. pallidum* genes in heterologous systems for antigenicity and immunogenicity screens. Functional studies on the haemolysins have shown that they do react weakly with preimmune sera but most of them do not show haemolysin phenotypes, so they may not be true haemolysins. Checking the source of this annotation uncovered a weak and unconvincing homology in the original assignment. They are also cloning and expressing all *T. pallidum* genes in various vector systems, as GST or His fusions. So far they have cloned 1008 and have 23 more to complete. They also plan to use some known antigens and some new potential antigens for phage display in rabbits. They have also tried large insert libraries in *E. coli*; they saw that at least half of the genes were expressed, so the promoters must have been pretty well conserved, despite the large evolutionary distance between them.

Copyright © 2002 John Wiley & Sons, Ltd.

### Organelle genomes, epigenetics and RNA

John Allen (Plant biochemistry, Lund University, Sweden) discussed **the function of cytoplasmic genomes**. He explained that it is well known that proteins are packed into organelles, with very little space left, so why have a genetic system, when space is at a premium? It is widely held that organelle genomes are a relic of their bacterial origin; most useful genes have been transferred to the nuclear genome, so why do chloroplasts and mitochondria retain a genome, when other compartments such as proteasomes do not? Why not move all the genes to the host genome? He detailed several explanations that have been suggested, including that of Bogorad [1] — so that the core components of multisubunit complexes can be synthesized *de novo* in the correct compartment. Herrman and Westhoff and others have suggested that it is because the evolutionary process of transferring genes to the nucleus is still incomplete, and von Heijne [7] suggested the related ‘frozen accident’ theory, that the evolutionary process of transfer was under way and something occurred that stopped it (although there is no evidence for this). Some discussions that have led to the suggestion that it is all a question of hydrophobicity, but there are no data to support this; another unattributed suggestion is that some proteins and co-factors cannot be imported and therefore must be encoded *in situ*. John Allen believes that it is because it allows redox control of gene expression, by which he means that the organelle-encoded genes control the ‘core’ assembly of complexes and need to be regulated *in situ* in response to the dangerous redox chemistry that goes on in the organelles. He thinks that the nuclear-encoded genes that are redox-regulated in plants and animals are regulated in a different way, and that the subunits encoded by these genes may be peripheral. This provides a reason why the mitochondria should come from the female, since this provides less chance for mutation and would be safer, with less risk of alteration in these dangerous genes (more details of John Allen’s discussion on the role of organelle genomes can be found in his review in our next issue).

Philip Avner (Institut Pasteur, France) talked about **a functional genomics approach to X-inactivation**. First he explained that epigenomics is the study of heritable, stable changes in genetic activity that are not based on changes in nucleotide sequence. Such effects are implicated in several inherited disorders and in the development of cancer. X-inactivation is the most extensive epigenetic mechanism known, which potentially controls 1500–2000 genes on the X chromosome. It is tightly coordinated with embryogenesis and the establishment of cell differentiation. It also implies that there must be recognition of different copies of the X chromosome in the cell. The inactive X is transcriptionally silent, and replicates late. It is depleted in acetylated forms of histones H3 and H4, and it has hypermethylated CpG islands. Inactivation occurs during early cell divisions of the embryo and is controlled by a master locus, the X inactivation centre (Xic). This unique region of the X chromosome is needed to ensure that only a single X is inactivated in each diploid female cell. It is responsible for choosing which copy is inactivated and for initiating the nucleation of silent chromatin on the chosen chromosome. The change from the active to the inactive state is associated with the accumulation of a large non-coding RNA (encoded by the Xist gene, located in the Xic region), which seems to spread in *cis* from Xic until it ‘decorates’ the entire inactive X. Silencing occurs rapidly after the initiation of inactivation and is associated with extensive modifications of the chromatin structure (which are, as yet, poorly characterized).

Genomic studies of the Xic region have identified several elements, including Xist, Tsix (a 40 Kb antisense transcript of Xist with a CpG island), Tsx, and a 5′ hotspot that is a region of possible chromatin nucleation. Female ES cells can be used as an *ex vivo* model system to study this mechanism, and they have tried deleting these elements in these cells. A 65 Kb deletion in the Tsix region increased the steady-state level of Xist, and resulted in the mutant copy being the chromosome chosen for inactivation; it affected the choice and counting stages of the process. A smaller deletion resulted in normal levels of Xist; the mutant chromosome was always chosen for inactivation, as before, and initiation of inactivation was repressed. They concluded that there are multiple control elements; neither choice nor counting are mediated solely by Tsix, but it does regulate the level of Xist. They have also looked at the 5′ region in relation to histone H3 methylation, since histone modification is thought to be associated with active and inactive states of

Copyright © 2002 John Wiley & Sons, Ltd. 

chromatin. They showed that a Lys9 methylated form of H3 is associated with promoters in the Xic region in females and that the amount of this form that is associated increases when Xist levels increase. The association of this form with the promoters is a late event, and early, global methylation of H3 Lys 9 must affect other loci. It could perhaps recruit Xist to sites along the chosen X. They have also looked at the Xic region of the mouse genome. They have found several protein-coding genes, four untranslated RNA genes and two retrotransposon genes. They see an evolutionary selection against insertion of LINEs in actively transcribed genes and widespread intergenic transcription of untranslated RNA. The Tsix sequence is not conserved in humans and neither are all the regulatory regions, so they can't predict the pattern of regulation. He suggested that the role of the non-translated RNA is likely to be critical for X inactivation.

Eric Westhof (ULP, Strasbourg, France) gave a talk on **structural bioinformatics of structured RNAs**. He explained that RNA motifs fold autonomously, and that they are recurrent and limited in number. Each RNA structure can be parsed into key motifs; the folding is determined by the laws of chemistry and physics. RNA motifs are conserved across the tree of life and are used for diverse functions. Watson–Crick (W–C) base pairs in RNA make the helices, which form a scaffold, but the non-W–C pairs dominate the tertiary structure, making the motifs, and are responsible for RNA–RNA recognition, etc.

He then described a proposed nomenclature for non-W–C pairs that avoids ambiguous, redundant, historical or contingent terms and allows for easy visualization of the base pair geometry. It also aids in identifying isosteric relationships and facilitates homology modelling. It treats bases as triangles and describes the interacting pairs by which of the three edges each base uses to make the interaction (W–C, Hoogsteen, or sugar edge), and the orientation of the bases — *cis* or *trans*. He has devised a series of symbols to denote the possible combinations, which can be used to draw diagrams and to more easily compare RNA structures (for more details of this nomenclature and its applications in the study of RNA structure, see Leontis and Westhof in this issue, p. 518).

Daniel Gautheret (University of Marseilles, France) gave a presentation entitled '**RNA bioinformatics: the identification of non-coding RNA in genomic sequences**'. He introduced the field of RNomics, which includes non-coding RNA (ncRNA), coding RNA with functional motifs, and introns. Non-coding RNA is defined by primary sequence and secondary structure. Using substitution matrices for nucleic acids to identify them works very poorly compared to using them to find coding DNA, as pairing of bases in the structure is more important, so base changes can happen. This means that BLAST will only find a few of them and hidden Markov modelling is not so good either. Despite this, all the tRNAs have so far been found by using BLAST. There are existing tools for tRNA prediction, for group 1 intron prediction and for snoRNA prediction, and there are flexible tools that use human made descriptors to do a fast database search (although these are time-consuming and provide only a yes/no answer, with no scoring). There is a need for probabilistic predictors; it is not enough to say that a base is paired as there are base pair biases that are linked to function, which could escape human inspection. There have been some studies using stochastic, context-free grammars, but these were not practical for large alignments or genome-wide searches and were costly in terms of time. His group have written ERPIN, a profile descriptor of sequence alignments that uses classic profiles and provides a Lod score with each prediction, and SECIS, a selenocysteine insertion sequence search tool, which has been trained on 43 selenoproteins. They used these tools together, iteratively, on ~4 Gb of sequence and predicted 120 sure SECIS hits and 200 potential hits (see **http://tagc.univ-mrs.fr/pub/erpin/**).

*De novo* prediction of ncRNAs is another challenge. Approaches tested so far include thermodynamic profiling (but this has only had very limited success) and G + C content, or a combination of G + C% and CpG%, which worked well only for high A + T content genomes. RNA Genie [2] was trained on *E. coli* DNA sequence, which was split into a negative set (intergenic regions) and a positive, true ncRNA, set. It uses a combination of parameters, including nucleotide and dinucleotide composition, to make predictions and the group claim 80–90% accuracy. Other groups have used comparative genomics to help find ncRNAs, such as the QRNA tool of Rivas and Eddy [3] and the study of mouse and human intergenic regions by Shabalina *et al.* [4].

Copyright © 2002 John Wiley & Sons, Ltd.

### References

1. Bogorad L. 1975. Evolution of organelles and eukaryotic genomes. *Science* **188**: 891–898.
2. Carter RJ, Dubchak I, Holbrook SR. 2001. A computational approach to identify genes for functional RNAs in genomic sequences. *Nucleic Acids Res* **29**(19): 3928–3938.
3. Rivas E, Eddy SR. 2001. Noncoding RNA gene detection using comparative sequence analysis. *BMC Bioinformatics* **2**(1): 8.
4. Shabalina SA, Ogurtsov AY, Kondrashov VA, Kondrashov AS. 2001. Selective constraint in intergenic regions of human and mouse genomes. *Trends Genet* **17**(7): 373–376.
5. TCDB: **http://www.biology.ucsd.edu/~msaier/transport**.
6. Vitkup D, Melamud E, Moult J, Sander C. 2001. Completeness in structural genomics. *Nature Struct Biol* **8**(6): 559–566.
7. Von Heijne G. 1986. Why mitochondria need a genome. *FEBS Lett* **198**: 1–4.

---

The Meeting Highlights of *Comparative and Functional Genomics* aim to present a commentary on the topical issues in genomics studies presented at a conference. The Meeting Highlights represent a personal critical analysis of the current reports, which aims at providing implications for future genomics studies.

---

Copyright © 2002 John Wiley & Sons, Ltd.
